# The Impact of Organic Farming on Quality of Tomatoes Is Associated to Increased Oxidative Stress during Fruit Development

**DOI:** 10.1371/journal.pone.0056354

**Published:** 2013-02-20

**Authors:** Aurelice B. Oliveira, Carlos F. H. Moura, Enéas Gomes-Filho, Claudia A. Marco, Laurent Urban, Maria Raquel A. Miranda

**Affiliations:** 1 Universidade Federal do Ceará, Depto. Bioquímica e Biologia Molecular, Fortaleza-CE, Brazil; 2 Embrapa Agroindustria Tropical, Fortaleza-CE, Brazil; 3 Universidade Federal do Ceará, Campus do Cariri, Av. Tenente Raimundo Rocha s/n - Cidade Universitária, Juazeiro do Norte-CE, Brazil; 4 Université d’Avignon et des Pays de Vaucluse, Campus Agroparc, Avignon, France; Cairo University, Egypt

## Abstract

This study was conducted with the objective of testing the hypothesis that tomato fruits from organic farming accumulate more nutritional compounds, such as phenolics and vitamin C as a consequence of the stressing conditions associated with farming system. Growth was reduced in fruits from organic farming while titratable acidity, the soluble solids content and the concentrations in vitamin C were respectively +29%, +57% and +55% higher at the stage of commercial maturity. At that time, the total phenolic content was +139% higher than in the fruits from conventional farming which seems consistent with the more than two times higher activity of phenylalanine ammonia lyase (PAL) we observed throughout fruit development in fruits from organic farming. Cell membrane lipid peroxidation (LPO) degree was 60% higher in organic tomatoes. SOD activity was also dramatically higher in the fruits from organic farming. Taken together, our observations suggest that tomato fruits from organic farming experienced stressing conditions that resulted in oxidative stress and the accumulation of higher concentrations of soluble solids as sugars and other compounds contributing to fruit nutritional quality such as vitamin C and phenolic compounds.

## Introduction

The consumption of fruit and vegetables has been associated with lower risk of chronic human health problems like cardiovascular diseases, cancer, hypertension and diabetes type two due to their high contents in dietary bioactive compounds, the so-called phytochemicals, endowed with protective properties [1]; [2]; [3]; [4]; [5]. Until recently, the health benefits of fruits and vegetables have been attributed to the antioxidant properties of the phytochemicals they provide. However, nowadays, the routine consumption of antioxidant supplements has become highly controversial as studies have demonstrated that they can actually be harmful [6]. Besides the antioxidant theory, there are now alternative theories about the way phytochemicals induce protective mechanisms in consumers. For instance, it has been shown that a large range of secondary metabolites in fruit and vegetables as phenolic compounds [7] act as elicitors that activate Nrf2, a transcription factor that binds to the antioxidant response element in the promoter region of genes coding for enzymes involved in protective mechanisms [4]. Supplementation of processed tomato products, containing lycopene, has shown to lower biomarkers of oxidative stress and carcinogenesis in healthy and type II diabetic patients, and prostate cancer patients, respectively. The mechanisms of action involve protection of plasma lipoproteins, lymphocyte DNA and serum proteins against oxidative damage, and anticarcinogenic effects, including reduction of prostate-specific antigen, up-regulation of connexin expression and overall decrease in prostate tumor aggressiveness [5].

As stressed in a recent survey of literature, environmental factors represent a powerful lever to increase the concentrations in phytochemicals [8]. Among all the factors that seem effective in enhancing the concentrations in phytochemicals in fruits and vegetables, stress emerges as especially promising. This makes sense considering that all stresses, either biotic or abiotic, are conducive to oxidative stress in plants [9] and that oxidative signaling controls synthesis and accumulation of secondary metabolites [10]; [11]; [12]. Thereby, it may be hypothesized that cropping systems that allow plants to undergo (moderate) stress such as organic farming result in products with higher concentrations in phytochemicals resulting of low mineral availability and, therefore of the diversion of carbon skeletons from protein synthesis [13]. Indeed, several studies have demonstrated that fruits and vegetables from organic farming generally are endowed with enhanced nutritional properties [14]; [15]; [16]; [17]. A recent comparative study showed that organic tomato juice has a higher phenolic content and hydrophilic antioxidant activity when compared to conventional tomato juice [17]. Organic tomatoes from Felicia, Izabella and Paola varieties had higher vitamin C and carotenoid contents which were more pronounced when expressed on fresh matter than on dry matter [14]. Organic strawberries present higher antioxidant concentrations and have been shown to inhibit the proliferation of human colon (HT29) and breast (MCF-7) cancer cells more effectively than conventional ones [15].

The hypothesis that oxidative stress is involved in enhanced concentrations in phytochemicals of fruits and vegetables from organic farming has rarely been tested to our knowledge [18]. The objective of this paper is to contribute to fulfilling this gap by comparing not only the concentrations in compounds contributing to quality in fruits from organic and conventional farming, but also by measuring indicators of oxidative stress, namely the activities of antioxidant enzymes, superoxide dismutase (SOD), ascorbate peroxidase (APX) and catalase (CAT), the concentration in ascorbate (AsA) and cell membrane lipid peroxidation (LPO) degree. The study was conducted on tomatoes which are climacteric fruits, representing a relevant source of vitamins C and E and other phytochemicals such as carotenoids and polyphenols [19]. We focused in this study on phenolic compounds and the activity of phenylalanine ammonia-lyase (PAL) because this enzyme controls a rate-determining step of the biosynthetic pathway of phenolic compounds in plants and is well-known to be induced by environmental stress [20]; [21]; [22]; [23].

## Materials and Methods

### Fruit Material

Organic and conventional tomato fruits (*Solanum lycopersicum* cv. Débora) cultivated organically and conventionally were obtained from local producers of the district of Crato-Ceará State at northeastern part of Brazil (7°14′03′′S and 39°24′34′′W). The organic and conventional farms were within 1.5 km from each other, therefore with similar environmental conditions and plants were planted in rows 1 m apart with 0.4 m between plants. The soil was representative of this region of Brazil and classified as humic yellowish latosol with medium phosphate and potassium levels, medium-textured clay and 0–20 cm top layer with pH (H_2_O) 6.2; cation exchange capacity of (cmolc.dm^−3^) Ca^2+^2.0; Mg^2+^0.8; K^+^0.2; Al^3+^0.0; P (mg.dm^−3^) 0.6 and organic matter (g.kg^−1^) 2.6. As reported by the growers, the organic cultivation system used a compost of animal manure (10 t.ha^−1^), legume cocktail and sugar cane bagasse incorporated into soil just before sowing; and every 10 days, 0.5% Bordo mix (a mixture of copper (II) sulfate (CuSO_4_) and slaked lime) was used preventively as a fungicide. In the conventional system, the pesticide FASTAC^®^100 was applied at 0.01% when needed and inorganic fertilizer was used as recommended by the Brazilian agricultural development department at the following rates: 100 kg.ha^−1^ for nitrogen, 400 kg.ha^−1^ for P_2_0_5_ and 80 kg.ha^−1^ for K_2_0.

Organic and conventional tomatoes were evaluated at three developmental stages: immature (green colored), physiologically mature (breaker), and at the harvesting stage (red). Fruits were harvested manually from 30 plants for each system, then washed in tap water and carefully selected to insure good uniformity in maturity and size. After cleansing and selection, fruits from each treatment were divided into samples made up of four replicates each consisting of twelve fruits. Fruit pericarp was ground and homogenized using a domestic food processor and then stored at −20°C for further analysis.

### Fruit Quality Parameters

Weight was measured on a semi-analytical scale (TECNAL, São Paulo-Brazil) as whole fruits were individually weighed and results expressed in grams (g). Size measurements were made with a handheld pachymeter (ZAAS Precision, São Paulo-Brazil) and expressed in centimeters (cm). Soluble solids (SS) content was determined by refractometry as described by AOAC [24] using a digital refractometer (ATAGO N1, Kirkland-USA) with automatic temperature compensation. The results were expressed in °Brix (concentration of sucrose w/w). The pH was measured using an automatic pHmeter (Labmeter PHS-3B, São Paulo-Brazil) as recommended by AOAC (24). Titrable acidity (TA) was evaluated following AOAC (24) by using an automatic titrator (Mettler-Toledo DL12, Columbus-USA). Results were expressed as % of the predominant acid for each species.

### Antioxidants

The total antioxidant activity (TAA) was determined using the ABTS method as described by Re and others [25], which measures the ability of lipophilic and hydrophilic antioxidants to quench a 2,2′-azino-bis 3-ethylbenzthiazoline-6-sulphonic acid (ABTS^*+^, Sigma) radical cation. Before the colorimetric assay, the samples were subjected to a procedure of extraction in 50% methanol and 70% acetone as described by Larrauri and others [26]. The radical solution was formed using 7 mM ABTS^*+^ and 140 mM potassium persulfate, incubated and protected from light for 16 h. Once the radical was formed, the reaction was started by adding 30 *µ*L of extract in 3 mL of radical solution. Absorbance was measured (734 nm) after 6 min, and the decrease in absorption was used to calculate the TAA. A calibration curve was prepared and different Trolox (Sigma) concentrations (standard trolox solutions ranging from 100 to 2000 *µ*M) were also evaluated against the radical. Antioxidant activity was expressed as Trolox equivalent antioxidant capacity (TEAC), *µ*mol Trolox. g ^−1^ FW (fresh weight).

The total phenolic content was measured by a colorimetric assay using Folin–Ciocalteu reagent (Sigma) as described by Obanda and Owuor [27]. Before the colorimetric assay, the samples were subjected to a procedure of extraction in 50% methanol and 70% acetone as described by Larrauri and others [26]. Absorbance was measured at 700 nm, gallic acid (Acros Organics) was used as the standard and results were expressed as galic acid equivalents (GAE) mg.kg^−1^ FW.

Anthocyanins and yellow flavonoids were extracted and determined as described by Francis [28]. The absorbance of the filtrate was measured at 535 nm and at 374 nm for total anthocyanin and for yellow flavonoid content using absorption coefficients of 98.2 and 76.6, respectively. The results were expressed as mg. kg^−1^ FW.

Total vitamin C was determined by titration with Tillman solution (0.02% 2,6 dichloro-indophenol, DFI from Sigma) described by Strohecker and Henning [29]. The results were expressed as mg. kg^−1^ FW.

### Enzymes

Samples of two grams of pulp were homogenized in an ice-cold extraction buffer (100 mM potassium-phosphate buffer pH 7.0+0.1 mM EDTA). The homogenate was filtered through a muslin cloth and centrifuged at 3300×*g* for 40 min. The supernatant fraction was used as a crude extract for antioxidant enzyme activity assays. All the procedures were performed at 4°C. The total protein content was determined according to Bradford [30].

Catalase (CAT, EC 1.11.1.6) activity was measured according to Beers and Sizer [31]. The decrease in H_2_O_2_ (Merck) was monitored through absorbance at 240 nm and quantified by its molar extinction coefficient (36 M^−1^ cm^−1^). The results were expressed as *µ*mol H_2_O_2_. min^−1^. mg^−1^ P (protein).

Ascorbate peroxidase (APX, EC 1.11.1.1) activity was assayed according to Nakano and Asada [32]. The reaction was started by adding ascorbic acid and ascorbate oxidation was measured through absorbance at 290 nm. Enzyme activity was measured using the molar extinction coefficient for ascorbate (2.8 mM. cm^−1^) and the results expressed in *µ*mol H_2_O_2_. min^−1^. mg^−1^ P, taking into account that 1 mol of ascorbate is required for the reduction of 1 mol H_2_O_2_.

Superoxide dismutase (SOD, EC 1.15.1.1) activity was determined by spectrophotometry, based on the inhibition of the photochemical reduction of nitroblue tetrazolium chloride (NBT, Sigma) [33]. The absorbance was measured at 560 nm and one unit of SOD activity (UA) was defined as the amount of enzyme required to cause a 50% reduction in the NBT photo-reduction rate. Thus, results were expressed as UA. mg^−1^ P.

Phenylalanine ammonia-lyase (PAL, EC 4.3.1.24) activity was assayed with samples extracted by a modified version of the method developed by Mori and others [34]. Pulp samples (1 g) were homogenized for 3 min at 4°C with (2 mL) 0.1 M Tris-HCl buffer solution (pH 8.0), 1 mM EDTA and 0.5 g PVP, and then centrifuged at 5000×*g* for 20 min. The supernatant was the extract used to determine the PAL activity which was assayed using an assay modified from that of El-Shora [21]. The reaction mixture contained 100 mM Tris-HCl buffer (pH 8.4), 40 mM L-phenylalanine and 100 *µ*L of enzyme to a total volume of 880 *µ*L. The reaction was stopped by adding 6 M HCl and the *A*_290_ of the clear solution was measured. The PAL activity was expressed as *µ*mol of trans-cinnamic acid. mg ^−1^ P.

### Lipid Peroxidation Degree

The thiobarbituric acid reactive substances (TBARS) assay measures lipid peroxidation degree through determination of hydroperoxides and aldehydes such as malondialdehyde (MDA). MDA reacts with thiobarbituric acid (TBA, 1∶2) to form a fluorescent adduct MDA-(TBA)_2_ using a modified version of the method developed by Zhu and others [35]. Pulp samples (0.5 g) were homogenized in 5 mL of 0.1% trichloroacetic acid (TCA) and centrifuged at 3300×*g* at 4°C for 20 min. The supernatant (750 *µ*L) was collected and added to 3 mL 0.5% TBA in 20% TCA and incubated at 95°C for 30 min. Following incubation, the tubes were immediately cooled in ice bath and centrifuged at 3000×*g* for 10 min. Absorbance at 532 nm was measured and corrections were made for unspecific turbidity by subtracting the absorbance at 600 nm. TBARS are expressed as MDA equivalents, calculated using an extinction coefficient of 155 mmol.cm^−1^ and expressed as nmol. g^−1^ FW.

### Leaf Relative Chlorophyll Content

The relative levels of total chlorophyll were estimated with a portable chlorophyll meter (SPAD-502 Minolta, Osaka, Japan). Measurements were performed on four of the youngest fully expanded leaves on five different plants and results were expressed as SPAD values.

### Statistical Analysis

All results are expressed as means ± standard error (SE). Analysis of variance, followed by multiple comparisons of means was performed using SISVAR version 5.1. Means were compared using Tukey’s test at α = 0.05.

## Results and Discussion

### Fruits from Organic Farming were More Stressed than Fruits from Conventional Growing Systems

The quality parameters of organic and conventional tomatoes were evaluated throughout the period of maturation of fruits (Table 1). There were substantial differences in growth between the fruits of the two growing systems compared. Mass and size were about 40% higher in fruits from conventional growing systems than in fruits from organic farming. Such differences could originate either from differences in nitrogen availability or from limitations to growth imposed by the more stressing conditions prevailing in organic farming [13]. The idea that stressing conditions impact negatively fruit size and mass is a very common one. Zushi and others [36], for instance, observed that salt-stressed tomatoes had a shorter developmental period with lower mass and accelerated ripening.

**Table 1:** Changes in quality parameters and chlorophyll content of tomatoes cultivated organically (OG) and conventionally (CV).

Tomato							
Parameters	Stage		OG		CV		
Weight (g)	Immature		67.10±12.40 Bb		103.02±15.53 Ab		
	Mature		84.88±26.40 Ba		131.52±22.29 Aa		
	Ripe		75.15±7.24 Bab		124.93±41.85 Aa		
Width (cm)	Immature		4.68±0.33 Bab		5.16±0.51 Aa		
	Mature		5.14±0.82 Aa		5.58±0.55 Aa		
	Ripe		4.20±0.37 Bb		5.46±0.83 Aa		
pH	Immature		4.36±0.06 Ba		4.53±0.07 Aa		
	Mature		4.46±0.06 Aa		4.43±0.04 Aa		
	Ripe		4.39±0.10 Aa		4.50±0.07 Aa		
TA (% citric acid)	Immature		0.33±0.01 Ab		0.28±0.02 Ba		
	Mature		0.25±0.00 Ac		0.28±0.03 Aa		
	Ripe		0.36±0.00 Aa		0.28±0.00 Ba		
SS (°Brix)	Immature		4.80±0.17 Aa		4.17±0.15 Aa		
	Mature		5.03±1.06 Aab		4.20±0.10 Aa		
	Ripe		6.00±0.00 Aa		3.83±0.51 Ba		
Total phenolics (mg GAE.kg^−1^)		Immature		308.5±3.04 Ab		249.1±5.65 Aa	
		Mature		508.3±1.51 Aa		299.8±2.39 Ba	
		Ripe		556.5±5.40 Aa		232.5±0.62 Ba	
Anthocyanins (mg. kg^−1^)		Immature		5.1±0.10 Ba		8.0±0.19 Aa	
		Mature		2.5±0.05 Ba		9.0±0.16 Aa	
		Ripe		3.6±0.09 Ba		9.9±0.11 Aa	
Yellow Flavonoids (mg. kg^−1^)		Immature		27.8±0.15 Bb		37.4±0.33 Aa	
		Mature		26.1±0.33 Bb		33.3±0.43 Aab	
		Ripe		43.7±0.49 Aa		25.7±0.33 Bb	
Total Vitamin C (mg.kg^−1^)		Immature		134.1±0.20 Ac		89.4±0.05 Bb	
		Mature		220.5±0.12 Ab		175.3±0.20 Ba	
		Ripe		264.7±0.40 Aa		170.9±0.16 Ba	
Relative chlorophyll content				40.18±7.20 A		40.29±5.20 A	

Mean values in the same column followed by the same small letter did not differ significantly between the developmental stages; by Tukey’s test (*p* ≥ 0.05).

Mean values in the same line followed by the same CAPITAL letter did not differ significantly between the cultural systems, by Tukey’s test (*p* ≥ 0.05).

The hypothesis of lower nitrogen availability in organic farming cannot be totally excluded from our observations even though one would have expected substantial differences in leaf chlorophyll content, which was clearly not the case (Table 1). All the same, there are ample reasons to consider that, in our trial, conditions were more stressing in organic farming than in conventional growing systems. Superoxide dismutase (SOD) activity was significantly higher in organic tomatoes, especially at the harvesting stage (+90%), but not the activities of APX and catalase (Table 2). SOD scavenges radical superoxide by catalyzing its conversion to H_2_O_2_, which subsequently is neutralized by CAT or APX. It may, thus be hypothesized that in fruits from organic farming there was an increase in concentration in H_2_O_2_. This could explain the ca. 60% higher LPO degree (Table 2) and the up to 140% higher PAL activity (Table 2) we observed. The higher concentration in ascorbate (+57% at the stage of harvesting maturity) seems to confirm that oxidative stress was higher in fruits from organic farming than in fruits from conventional growing systems (Table 1). A higher concentration in ascorbate in fruits from organic farming seems consistent with other observations like the ones made by Chassy and others [37] on tomatoes.

**Table 2:** Metabolism parameters evaluated during the development of tomato cultivated organically (OG) and conventionally (CV).

Tomato				
Parameters	Stage	OG	CV	
Lipid Peroxidation (nmol MDA g^−1^ FW)	Immature	16.93±1.54 Aab	9.84±0.90 Ba	
	Mature	14.09±2.71 Ab	7.20±2.18 Aa	
	Ripe	19.24±0.09 Aa	8.06±0.65 Ba	
PAL Activity (*µ*mol cinnamic ac. g^−1^ mg^−1^ P)	Immature	6.72±0.88 Ab	2.54±0.24 Ba	
	Mature	8.22±0.67 Ab	4.06±0.24 Ba	
	Ripe	11.43±0.71 Aa	4.79±0.89 Ba	
Antioxidant Activity (*µ*M Trolox g^−1^ FW)	Immature	98.72±38.65 Aa	98.18±30.42 Aa	
	Mature	143.54±44.52 Aa	161.23±6.15 Aa	
	Ripe	128.34±22.89 Aa	136.28±57.54 Aa	
APX Activity (*µ*mol H_2_O_2_ g^−1^ min^−1^ mg^−1^ P)	Immature	143.54±44.52 Aa	161.23±6.15 Aa	
	Mature	128.34±22.89 Aa	136.28±57.54 Aa	
	Ripe	1.01±0.11 Aab	0.98±0.11 Aa	
CAT Activity (*µ*mol H_2_O_2_ g^−1^ min^−1^ mg^−1^ P)	Immature	4.27±0.20 Aa	4.78±5.22 Ab	
	Mature	3.90±1.63 Aa	8.28±4.41 Aab	
	Ripe	5.07±1.67 Ba	16.46±6.05 Aa	
SOD Activity (UA g^−1^ mg^−1^ P)	Immature	104.95±21.15 Aab	42.16±6.90 Ba	
	Mature	77.05±21.02 Ab	47.38±6.50 Aa	
	Ripe	121.76±8.33 Aa	22.27±10.08 Ba	

Mean values in the same column followed by the same SMALL letter did not differ significantly between the developmental stages; by Tukey’s test (*p* ≥ 0.05).

Mean values in the same line followed by the same CAPITAL letter did not differ significantly between the cultural systems, by Tukey’s test (*p* ≥ 0.05).

### The Metabolism of Phenolic Compounds was Stimulated in the Fruits from Organic Farming when Compared to Fruits from Conventional Growing Systems

Phenylalanine ammonia lyase (PAL) activity increased slightly during the development of tomatoes and was significantly higher (up to 140%) in organic fruits (Table 2). The total phenolic content differed greatly between cropping systems. Conventional tomato fruits presented lower and constant total phenol concentrations during their development (∼250 mg GAE. kg^−1^), while there was an increase from 308 to 556 mg GAE.kg^−1^ in organic fruits. PAL is a key enzyme in both plant development and defense [22]. It catalyzes the conversion of L-phenylalanine into transcinnamate, the initial committed step of the multi-branched phenylpropanoid pathway in higher plants. This step is known to be a rate-limiting one of the biosynthetic pathway of phenolic compounds; which explains why the concentration in total phenolic compounds was eventually higher during maturation of tomato fruits from organic farming (Table 1). Considering the stimulating role stress exerts on PAL activity [22]; [38]; [39], it is tempting to link the increase in PAL activity and the subsequent increase in total phenolics we observed in fruits from organic farming with the indicators of oxidative stress we measured. Several other studies have demonstrated that fruits and vegetables from organic farming are usually richer in phenolic compounds than those from conventional growing systems [18]; [20]; [23]. What is novel in our study is that our observations seem to confirm the hypothesis of a link between phenolic metabolism and oxidative stress.

Interestingly, yellow flavonoids and anthocyanins did not follow the pattern of total phenolics (Table 1). For instance, the concentration in yellow flavonoids was +70% higher in organic fruits when compared to fruits from conventional growing system, but only at the harvesting stage, which is consistent with similar observations by Mitchell and others [40]. The concentration in anthocyanins was lower in the fruits from organic farming at all three stages of fruit development. These discrepancies indicate that organic farming had the effect of modifying the levels of transcripts or the activities of enzymes controlling intermediary steps of the biosynthetic pathway of phenolic compounds. In spite of the changes in antioxidants, the total antioxidant activity was not significantly different among the organic and conventional tomatoes (Table 2).

### Our Observations Only Partly Support the Growth Differentiation Balance Hypothesis

It has often been hypothesized that high carbohydrate supply, that would result of low nitrogen availability and, therefore of the diversion of carbon skeletons from protein synthesis, is favorable to the synthesis of secondary metabolites through its positive influence on precursor availability [41]. But then, ecological theories, such as the much-debated Growth Differentiation Balance Hypothesis (GDBH) predict that at the highest levels of mineral resource availability, plants would decrease their relative investment in the so-called differentiation processes, including the secondary metabolism [42], [43], [44]. Indeed, our observations apparently support the GDBH. In conventional fruits, that correspond to the highest availability of mineral resources as mass and size data would suggest, the concentration in total phenolics was the lowest, in contrary of those found for fruits from organic farming (Table 1). As suggested by observations made on Citrus fruits [45], the primary/secondary metabolisms interaction/competition theory may apply to whole plants which have to continuously arbitrate between growth and defense, but is probably not relevant for storage organs like fruits where secondary metabolites accumulate in response to a development programme aiming, among others, at advertising its nutritional status to potential seed disseminators.

## Conclusions

Our work clearly demonstrates that tomato fruits from organic farming have indeed a smaller size and mass than fruits from conventional growing systems, but also a substantially better quality in terms of concentrations in soluble solids and phytochemicals such as vitamin C and total phenolic compounds. Until recently, the focus has been mainly on yield rather than on gustative and micronutritional quality of fresh plant products. This might be all right for staple food, but, as far as fruits and vegetables are concerned, it may be argued that gustative and micronutritional quality matter more than energy supply. Our observations suggest that, at least for fruit and vegetable production, growers should not systematically try to reduce stress to maximize yield and fruit size, but should accept a certain level of stress as that imposed by organic farming with the objective of improving certain aspects of product quality. More research is needed in the future to better understand the links between stress and oxidative stress, on one side, and oxidative stress and secondary metabolism in fruits, on the other side. Also the physiological mechanisms behind the positive effect of organic farming on fruit quality will require additional studies to be conducted.
